# Developing Substance Use Programming for Person-Oriented Recovery and Treatment (SUPPORT): protocol for a pilot randomized controlled trial

**DOI:** 10.1186/s40814-017-0212-1

**Published:** 2017-12-15

**Authors:** Dennis P. Watson, Bradley Ray, Lisa Robison, Huiping Xu, Rhiannon Edwards, Michelle P. Salyers, James Hill, Sarah Shue

**Affiliations:** 1 Department of Social and Behavioral Sciences, Indiana University Fairbanks School of Public Health, 1050 Wishard Blvd, Indianapolis, IN 46202 USA; 2 School of Public and Environmental Affairs, Indiana University-Purdue University Indianapolis, 801 W. Michigan St., Indianapolis, IN 46202 USA; 3 Department of Biostatistics, Indiana University Fairbanks School of Public Health, 410 W. 10th St., Indianapolis, IN 46202 USA; 4 Public Advocates in Community Re-Entry, 2855 N. Keystone Ave, Indianapolis, IN 46218 USA; 5 Department of Psychology, Indiana University-Purdue University Indianapolis, 420 N Blackford St., Indianapolis, IN 46202 USA; 6 Department of Computer Science, Indiana University-Purdue University Indianapolis, 402 N. Blackford, Indianapolis, IN 46202 USA; 7 School of Health and Rehabilitation Sciences, Indiana University-Purdue University Indianapolis, 1050 Wishard Blvd, Indianapolis, IN 46202 USA

**Keywords:** Substance use disorder, Substance abuse, Criminal justice, Re-entry, Recovery, Recovery-oriented system of care, Pilot randomized control trial

## Abstract

**Background:**

There is a lack of evidence-based substance use disorder treatment and services targeting returning inmates. Substance Use Programming for Person-Oriented Recovery and Treatment (SUPPORT) is a community-driven, recovery-oriented approach to substance abuse care which has the potential to address this service gap. SUPPORT is modeled after Indiana’s Access to Recovery program, which was closed due to lack of federal support despite positive improvements in clients’ recovery outcomes. SUPPORT builds on noted limitations of Indiana's Access to Recovery program. The ultimate goal of this project is to establish SUPPORT as an effective and scalable recovery-oriented system of care. A necessary step we must take before launching a large clinical trial is pilot testing the SUPPORT intervention.

**Methods:**

The pilot will take place at Public Advocates in Community Re-Entry (PACE), nonprofit serving individuals with felony convictions who are located in Marion County, Indiana (Indianapolis). The pilot will follow a basic parallel randomized design to compare clients receiving SUPPORT with clients receiving standard services. A total of 80 clients within 3 months of prison release will be recruited to participate and randomly assigned to one of the two intervention arms. Quantitative measures will be collected at multiple time points to understand SUPPORT’s impact on recovery capital and outcomes. We will also collect qualitative data from SUPPORT clients to better understand their program and post-discharge experiences.

**Discussion:**

Successful completion of this pilot will prepare us to conduct a multi-site clinical trial. The ultimate goal of this future work is to develop an evidence-based and scalable approach to treating substance use disorder among persons returning to society after incarceration.

**Trial registration:**

ClinicalTrials.gov (Clinical Trials ID: NCT03132753 and Protocol Number: 1511731907). Registered 28 April 2017.

## Background

Substance use is a growing concern in the US criminal justice system, as incarcerated adults and those on community supervision have significantly higher rates of substance use disorder (SUD) than the general population. Indeed, over half of all inmates meet the criteria for drug dependence, and nearly three quarters report using drugs regularly prior to incarceration [1–3]. While many prisons offer some type of treatment, as much as 85% of the inmate population with SUD never receive clinical services [2, 4]. Moreover, SUD treatment offered to inmates is rarely evidence-based and is therefore insufficient to address needs of those with the most severe substance use issues [5–7]. SUD has been demonstrated to negatively impact a number of outcomes for inmates after release from prison including mental and physical health and criminal recidivism [5, 8–11].

The vast majority of criminal justice interventions for SUD are aimed at offenders prior to incarceration (e.g., drug treatment courts) and do not fit within a recovery paradigm [5, 9, 11]. Recovery-oriented service models emphasize such factors as empowerment and consumer choice [12, 13], and there is evidence that behavioral health services emphasizing these factors lead to positive outcomes for clients [14–17]. Compatible with the recovery paradigm, though not well tested to date, the recovery-oriented system of care (ROSC) approach for returning inmates offers clients a choice in service participation and aims to strengthen both internal and external aspects of recovery capital [18, 19]. A ROSC provides client-focused, strength-based addiction care through a network of comprehensive treatment and recovery supports [18, 20], which make long-term recovery more likely [20, 21]. Support services often include nonclinical services (e.g., peer mentoring, support groups, employment assistance, and/or housing services) aimed at developing recovery capital through a holistic approach that considers the individual, family, and community [22, 23]. Support services can be driven by peers or professionals and offer a solution to the lack of chronic care models by supporting self-management and sustainable treatment [24]. By merging recovery support with client choice, a ROSC facilitates reduced substance use and abstinence through individualized treatment plans that address clients’ developmental stages of recovery and systematic needs [20]. Ultimately, this approach serves as a system-level solution within a community or state that aims to help clients not only achieve abstinence but to make significant progress in other areas of life [25], as the RSOC model is well-suited to develop sustainable supports and life-long skills that are transferable to natural settings and promote personal development [19, 26].

Based on a ROSC model, Substance Use Programming for Person-Oriented Recovery and Treatment (SUPPORT) was developed in response to the need for more comprehensive post-release substance use services. SUPPORT is based on Indiana’s Access to Recovery (IN-ATR) program, which was closed in 2014 after 7 years due to lack of continued federal backing [27, 28] despite local evaluation results demonstrating improvement in client recovery outcomes [29–31]. SUPPORT’s goals closely mirror IN-ATR in that they aim to develop flexible, comprehensive, and client-centered recovery services. The primary mechanisms through which SUPPORT will accomplish its goals include (1) services delivered by a certified peer recovery coach (i.e., person with lived experience in recovery who assists others in their recovery), (2) recovery-focused treatment plans developed around each client’s chosen goals, and (3) payment vouchers clients can use to access support services to meet their goals. Differentiating SUPPORT from its predecessor program IN-ATR, it will be administered by a community-based entity (rather than the state) and will focus exclusively on returning inmates within 3 months of prison release (IN-ATR had a wider eligibility that included pregnant women, military personnel, and methamphetamine users). Additionally, services will be delivered for a full year (ATR was 6 months) and by a certified peer recovery coach. Finally, vouchers will be able to cover a wider array of supports and services than they were in IN-ATR. These modifications are based on both the need to develop SUPPORT without government backing and lessons learned from the evaluation of IN-ATR.

**Fig. 1:**
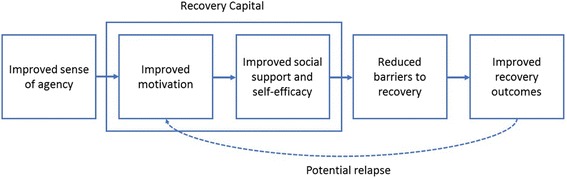
Model of expected client recovery process in the SUPPORT program. By increasing options available to clients through its expanded infrastructure and flexible services, the program improves client’s sense of agency (i.e., control) over their recovery. Increased agency improves motivation to participate in treatment and supportive services, as well as other aspects of recovery capital (e.g., social support and self-efficacy). Improved social capital reduces barriers to recovery and leads to improved recovery outcomes. The model also asserts that improved recovery capital and services support the individual through a relapse (should one happen) and reflects process-based definitions central to current recovery policy.

Figure 1 presents a model demonstrating how SUPPORT is expected to affect outcomes. By increasing options available to clients through its expanded infrastructure and flexible services, the program improves a clients’ sense of agency (i.e., control) over their recovery. The increased agency thus improves motivation to participate in treatment and supportive services, as well as other aspects of recovery capital (e.g., social support and self-efficacy). Improved social capital reduces barriers to recovery and leads to improved recovery outcomes. The model also asserts that improved recovery capital and services should support the individual through a relapse (should relapse happen) and reflects process-based definitions central to current recovery-oriented policy [13, 32]. Though it is clear several influences converge to determine outcomes of SUD treatment, there is significant evidence to confirm mainstream addiction models face limitations in design and fail to provide treatment options to support sustained recovery management, particularly for those reentering the community following incarceration [33–35].

The need for development and research related to the ROSC approach is highlighted by the significant lack of peer-reviewed research conducted on ATR despite the more than $200 million that was spent on the program. Those peer-reviewed studies that have been published demonstrate improvements in such factors as substance use, SUD treatment retention, recidivism, and Medicaid savings [31, 36, 37]. While this is encouraging, these studies—including the evaluation of IN-ATR [31]—were post hoc and observational in their approach, making it difficult to determine the extent to which ATR was directly responsible for outcomes. The current study seeks to develop a stronger evidence base for ROSC programs through a randomized trial of SUPPORT. The pilot described below is our first step in this process.

## Methods

This pilot study will follow a parallel, randomized design comparing SUPPORT to treatment as usual (TAU) [38]. The pilot is part of a larger study with additional development and feasibility aims, which are not described here. The primary goals of the pilot are to (a) establish feasibility of instruments and protocols, (b) obtain necessary information to determine the sample size for the subsequent larger trial through comparisons between SUPPORT and TAU client outcomes, and (c) identify key program elements and processes to assist us in hypothesis refinement to guide future work.

### Study setting

The pilot will be conducted at Public Advocates in Community Re-Entry (PACE), a nonprofit that provides services to individuals with felony convictions in Marion County, Indiana—the largest county in the state and home to Indianapolis, the state capital. PACE has provided re-entry services for over 55 years. Their services are divided into four distinct categories: transitional, employment, addiction, and pre-release services. It is the only former IN-ATR agency in Marion County specializing in a re-entry population.

### Eligibility criteria

All clients who are over the age of 18, who have a SUD, are within 3 months of prison release, and are no longer incarcerated (in a prison, jail, or work release facility) will be eligible for study participation. Sex offenders will be excluded from the pilot, as they face greater barriers to community integration and experience higher levels of supervision while on parole, which have the potential to confound results during a small-scale pilot.

### Intervention arms

**Fig. 2:**
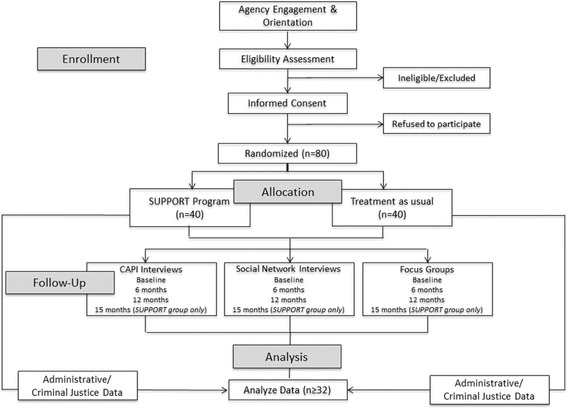
Flow of clients through the study. The process that clients will undergo to participate in the study, from eligibility to informed consent to randomization

Clients will be assigned to one of the two groups: (1) SUPPORT or (2) TAU. Figure 2 depicts how clients will move through the intervention arms.

#### Support

Clients assigned to the experimental group will be offered 12 months of SUPPORT services at PACE with a recovery coach. The recovery coach will guide the SUPPORT client through their recovery, offering guidance and support, while coordinating their treatment services, including support services. The SUPPORT program will provide clients with up to $700 worth of vouchers (depending on client’s stated goals and identified barriers) to cover the cost of the additional flexible support services over the 12 months of program enrollment. Additional voucher funding, up to $300, will be available to the client at the request of the recovery coach should additional resources be deemed necessary for them to meet the goals of their recovery plan. Vouchers can be used to cover support services in the following areas: housing (permanent and transitional), employment services (training, placement, and readiness), substance use treatment, transportation, childcare, educational or vocational services, and aftercare planning. Clients will not be responsible for keeping track of their vouchers; rather, the recovery coach will track all voucher spending and will assist the client in choosing appropriate services and monitoring service completion.

#### Treatment as usual

Clients in the TAU group will have fewer options than those in SUPPORT; specifically, they will not receive vouchers to access support services such as transportation and housing and will only receive standard case management and substance use counseling, which is more prescriptive and less intensive than the recovery coach services provided through SUPPORT.

### Measures

Demographic and background information on all clients will be collected at baseline, including date of birth, gender, race/ethnicity, sexual orientation, housing history, education, employment history, past involvement in addiction recovery and treatment, and criminal history. Data reflecting the constructs agency/self-determination, recovery capital (treatment motivation, social support/networks, and self-efficacy), and recovery-related outcomes (as depicted in Fig. 1) will also be collected.

We will administer a six-question subscale from the *Self-Determination Scale* to measure agency and self-determination. The subscale measures perceived choice by presenting subjects with two opposing statements labeled “A” (e.g., “I always feel like I choose the things I do.”) and “B” (e.g., “I sometimes feel that it’s not really me choosing the things I do.”). The subject is asked to rate which question they agree with more using a Likert scale ranging from 1 (only “A” feels true) to 5 (only “B” feels true). The introduction to the instrument will be modified to instruct clients to answer questions as they relate to their recovery treatment and services. The instrument has demonstrated reliability and predictive validity [39].

Treatment motivation will be measured using the *Treatment Motivation Questionnaire*, a 26-item instrument with demonstrated reliability and construct validity [40]. The questionnaire measures the attitudes about treatment and reasons for entering the treatment by asking the respondent to indicate how true each statement is (1 = not at all true to 7 = very true); the statements are grouped into three subscales: external reasons, internal reasons, and help-seeking.

Social support/networks will be measured using the *Health Matters Social Network Battery*, which contains eight questions that generate names of subjects’ social networks with whom they discuss important matters and health [41]. We will develop a third name-generating question to elicit names of individuals the respondent speaks to regarding substance use and substance use recovery. Data on other characteristics and attributes of persons named in the generator will also be collected (e.g., basic demographics, relationships between network members, and drug use attitudes and behaviors).

Finally, self-efficacy will be measured using the *General Self-Efficacy Adult Protocol*, which is a ten-item instrument measuring the degree to which individuals feel they can overcome challenges and accomplish tasks/goals in their lives. Items are scored on a 4-point scale (1 = not at all true to 4 = exactly true) and summed to yield the final composite score with a range from 10 to 40 [42]. The measure has been widely used for over 2 decades and has demonstrated consistent reliability [43].

### Outcome measures

We will use several measures to capture a variety of recovery-related outcomes. Our primary outcomes of substance use and abstinence will be measured using 12-items from the *National Survey on Drug Use and Health* [44] on the frequency of use for tobacco, alcohol, sedative, tranquilizers, painkillers, stimulants, marijuana, cocaine, crack, hallucinogens, inhalants, heroin, and prescription medications. Regarding secondary outcomes, to measure incremental progress toward recovery, we will use the *Stages of Change Readiness and Treatment Eagerness Scale* (SOCRATES), a 19-item instrument that assesses readiness to change behaviors in relations to substance use [45] by asking respondent to indicate level of agreement from 1 (No! strongly disagree) to 5 (Yes! strongly agree). The instrument has demonstrated reliability and convergent and predictive validity [46, 47]. To measure quality of life, we will use, the *current quality of life scale*, developed by the US Centers for Disease Control and Prevention, which consists of four questions aimed at measuring a respondent’s perceived health-related (both mental and physical) quality of life in the past 30 days [48].

Administrative data will capture several other secondary outcomes. To measure criminal history, we will capture data on all arrests, convictions, and periods of incarceration beginning 1 year before the client’s enrollment in the study through the end of the project. This will include county-level jail data and statewide prison records. Specific information collected will include a description of the crime committed to determine crime types (e.g., person, property, and drug-related), dates of each offense, and dates for commitment and release from incarceration in either jail or prison. We will also use PACE’s administrative data to capture housing status/stability, education/employment, income, physical and mental health status, and self-reported attendance at faith-based and self-help recovery groups as additional recovery-related outcomes.

Finally, we will also collect qualitative data from SUPPORT participants through focus groups aimed at developing an understanding of clients' experience of SUPPORT, and how it may have assisted them in overcoming barriers to recovery to assist us in further verifying the theoretical model depicted in Fig. 1.

### Sample size

A total of 80 clients will be recruited for participation in this pilot, which is slightly above the high end of the range of recommendations for a minimally acceptable pilot sample [49–51]. Participants will be randomized 1:1 to the SUPPORT pilot and treatment as usual, resulting in approximately 40 clients randomized to SUPPORT and 40 to usual care. The pilot sample size was determined to have a margin of error of 4 days for the estimation of the difference in the number of days of illicit drug use (our primary outcome) between subjects in the SUPPORT program and those in the usual condition after 1 year of SUPPORT programming at the 95% confidence level using the two independent sample *t* test. This sample size calculation was based on a conservative assumption that the standard deviation of days of illicit drug use at baseline is 9, which was derived based on the pilot data that show a standard deviation of 8.4 days at baseline.

### Recruitment

Trained research assistants will recruit clients for the participation during PACE’s standard orientation. Once research assistants determine a client’s eligibility, they will inform them of the opportunity to participate in the pilot study, describe the SUPPORT program services and, for those who agree to participate, obtain informed consent.

### Confidentiality

To protect confidentiality, we will assign each participant a subject identification number that will only be connected to their name on a file on the first author's personal server. The identification number will be used on all data collection components, including questionnaires, qualitative transcriptions, and criminal records. All completed informed consent documents and audio will be stored separately in a locked file cabinet in a locked office. All electronic data (quantitative and qualitative transcriptions) will be stored on a secured network and server maintained by the research staff behind a university firewall.

### Group assignment

We will use a random number generator to develop a pre-established list that will determine which intervention arm a client is placed in based on the point at which they enter the program [52]. Prior to meeting clients for enrollment, research assistants will receive data collection packets labeled with a pre-established identification number and containing a concealed card with the group assignment that has been randomly pre-assigned to the identification number. After the baseline assessment to determine eligibility, the client will be asked to consent to participate in the study; if they consent, the research assistant will open the envelope containing the card and notify the client of their group assignment.

### Data collection procedures

In addition to obtaining consent, research assistants will collect data using a computer-assisted personal interview (CAPI) [53, 54] (i.e., individual interviews assisted by computer technology) in the Research Electronic Data Capture (REDCap) system [55]. Each research assistant will have a tablet computer to access the interview. The research assistant will sit with the participant in a private room and read all questions out loud. The research assistant will collect all necessary demographic and service data, as well as the questions related to agency/self-determination, treatment motivation, self-efficacy, substance use frequency, and quality of life. At the beginning of the interview, the research assistant will remind the participant that the researchers are the only ones who will have access to their answers to these questions. Interviews will be conducted at baseline and 6 and 12 months to understand the change in outcomes over time. For the SUPPORT group only, research assistants will conduct an additional interview at 15 months to understand retention of treatment effects 3 months post-discharge. This process is estimated to take between 60 and 90 min. We will provide all clients with $30 for each interview. Should a client recidivate while enrolled in the study, no data collection will occur while the client is incarcerated. Clients will also be entered into a drawing for one of the two $100 visa gift cards at each data collection point.

Structured social network interviews will also be conducted by a trained research assistant at the time of enrollment and again at 6 and 12 months. For the SUPPORT group only, research assistants will conduct an additional social network interview at 18 months. The total data collection time for these interviews will be between 45 and 60 min depending on the number of individuals in the client’s network. Clients will receive $30 for taking part in each of these interviews. Research team members will also conduct focus groups with SUPPORT clients after their 15-month CAPI. We will conduct between five and eight focus groups with five to ten participants each. Focus groups will last between 60 and 90 min and will be audio-recorded. We will pay the client $30 for each focus group participant. Finally, we will work with our partner organizations’ administrators to identify and collect any administrative data not collected in the CAPI interview system and link it with data in the REDCap system (Table 1).

**Table 1:** Data collection schedule for participants

Data collection method	Measures	Enrollment	6 months	12 months	15 months
Structured interview (CAPI)	Recovery-related outcomes	All participants	All participants	All participants	SUPPORT group only
Structured interview	Social networks	All participants	All participants	All participants	SUPPORT group only
Recidivism and administrative data pulls	Public records	All participants	All participants	All participants	All participants
Focus groups	Qualitative data	n/a	n/a	n/a	SUPPORT group only

Research assistants will screen clients, consent them, assign them to SUPPORT or comparison groups, and collect data using a computer-assisted personal interview at baseline and 6 and 12 months to understand the change in outcomes over time. Research assistants will also conduct an interview at 15 months with clients assigned to the SUPPORT group to understand retention of treatment effects 3 months post-discharge. Researchers will conduct structured social network interviews within 1 week of the client’s entrance in the study and at 12 months. We will conduct focus groups with SUPPORT clients after their 15-month interview. We will conduct between five and eight focus groups with five to ten participants each. We will attempt to recruit all SUPPORT clients for focus groups. We will collect publically available data on recidivism from two websites operated by the Indiana Department of Corrections and the Marion County Jail using subjects’ name and date of birth and work with PACE administrators to identify and collect any service data not collected in the interview system and link it with data system

### Client retention

The research team will work with PACE staff to locate and retain clients in the study. PACE retained and was able to complete federally required data collection for over 80% of the IN-ATR clients they served, which is sufficient for our purposes. Research assistants will contact these individuals to schedule their CAPI and social network interviews and make sure they are completed.

### Data analysis

We will calculate the acceptance rate for all eligible individuals asked to participate in the study and the retention rate for all study participants. Mean and standard deviation will be computed for continuous variables and frequency, and the proportion will be computed for categorical variables. Due to the pilot nature of the study, we will focus on confidence interval estimation related to outcomes, and all hypothesis testing regarding group differences will be exploratory [56]. The effect of SUPPORT on client recovery outcomes will be summarized using 95% confidence intervals. In addition, we will use the mixed-effects model, where measures at follow-up visits are considered as the dependent variable, and the baseline outcome measure will be adjusted as a covariate. The clustering effect of repeated measures within a subject will be accommodated using a subject-specific random effect. We will also include time of measurement and treatment group in the model, as well as the interaction between these two variables to allow differential longitudinal patterns for subjects receiving the SUPPORT program and those receiving TAU. Time of measurement will be considered as a categorical variable to accommodate the possible nonlinearity of the longitudinal pattern. Effect of the SUPPORT program relative to TAU will be estimated based on the model. We choose to use the mixed-effects model because of its flexibility in handling repeated measures and missing data. We will perform sensitivity analysis to examine the extent to which results are affected by the missing data. Analysis based on complete cases, last observation carried forward (implying no change over time), and mean imputation will be performed. We will compare the results based on these models to the mixed-effects model results.

Our analysis of social network data will focus on the overall characteristics of the network (e.g., density and centrality) and the quality of different relationships in the network [57, 58]. We will carry out the first step of this analysis in EgoNet network analysis software [59], a software package for the analysis of social network data. In a second step, we will compare the outcomes for each of the network variables for the two groups. Regarding recidivism, we conduct pre-posttests to look at changes in number of days clients spent incarcerated prior to the study enrollment to post-enrollment. For this analysis, we will examine 6- and 12-month pre-post for both the treatment and control group. Using Fisher’s exact test and between-group tests, we will consider whether there are differences in the likelihood of any criminal recidivism between the treatment and control group. We will also estimate event history models using Cox regression survival analysis to model the time to recidivism while controlling for covariates and repeated measures of various intermediate and collateral outcomes to consider the impact of participation in the program and of treatment characteristics on the likelihood of and length of time to recidivism. Finally, we will also be examining the differences between the TAU and SUPPORT group on our primary outcome variable (substance use), as well as other recovery measures, and will use similar models as those noted above to conduct subgroup analysis to explore associations to our main outcome measures.

Analysis of focus group data follows a method of inductive coding outlined by Thomas where themes are identified as they pertain to the primary research questions [60]. We will determine saturation at the point when there is no longer any incremental learning in relation to the research questions as we move between data and theory [61]. We will use MAXQDA qualitative data analysis software to facilitate the analysis process [62]. As a final step in the analysis, we will triangulate qualitative findings and quantitative results to enhance their validity and assist in refining our hypotheses for the subsequent trial [56, 63].

### Data safety and monitoring plan

To protect patient confidentiality and ensure participant safety, we will hold monthly meetings with PACE staff and conduct preliminary analyses of the data (at 6, 12, and 15 months) to monitor client withdraws and complaints, as well as to ensure participant safety and identify any significant negative outcomes or unintended consequences associated with SUPPORT involvement or the research protocols. The Principal Investigators will verify the accuracy and completeness of data collection and safety reports throughout the entire study period.

## Discussion

There is currently a lack of sufficient community-based SUD interventions tailored for returning inmates that provide support services necessary for overcoming barriers associated with re-entry (e.g., food, transportation, clothing, and housing) [64]. Indeed, there are currently no interventions listed within the National Registry of Evidence-Based Programs and Practices targeting this population [65]. This pilot is a first step in developing an effective ROSC model for SUD treatment and services for returning inmates. The development of such an intervention is also in the best interest of policymakers, providers, and clients. While national and state policies have recently moved toward criminal justice reforms encouraging implementation of a continuum of recovery-oriented, evidence-based interventions in the criminal justice system [66, 67], there remains a lack of adequate programming for returning inmates with SUD. Additionally, the project will increase general scientific knowledge related to recovery-oriented services—an area of research that is currently lacking [68–70]—as well as provide justification for the expenditure of resources on other ROSC models to prevent them from suffering the same fate as ATR.

Assessment of the feasibility of protocols to advance to the subsequent trial will hinge on our ability to obtain a study recruitment rate above 60% and a participant retention rate above 70% (goals based on our prior experience the IN-ATR program, as well as our expectation that potential modifications to improve participant satisfaction with the study and intervention protocols will be needed based on learning during the pilot). Following this pilot, semi-structured phone interviews will be conducted with potential adopters of SUPPORT to identify possible barriers and facilitators to the scalability of the intervention [71]. This will help prepare us for a larger trial that will include multiple service organizations and a more diverse group of subjects (e.g., inclusion of sex offenders). This future work will also include aims investigating implementation and service outcomes that will be necessary for developing strong dissemination and implementation strategies should SUPPORT be demonstrated effective.

## Trial status

This manuscript was originally submitted for peer review prior to any client recruitment. Client recruitment began on October 23, 2018, and nine clients have been recruited for this pilot as of November 8, 2017.

## Abbreviations

CAPI: Computer-assisted personal interview
IN-ATR: Indiana Access to Recovery
PACE: Public Advocates in Community Re-Entry
REDCap: Research Electronic Data Capture System
ROSC: Recovery-oriented system of care
SUD: Substance use disorder
SUPPORT: Substance Use Programming for Person-Oriented Recovery and Treatment
TAU: Treatment as usual
